# Drp1 Regulated Mitochondrial Hypofission Promotes the Invasion and Proliferation of Growth Hormone-Secreting Pituitary Adenomas *via* Activating STAT3

**DOI:** 10.3389/fonc.2022.739631

**Published:** 2022-04-07

**Authors:** Yin Zhang, Lei Zhang, Kexia Fan, Yajun Gou, Zhenle Zang, Xiao Ding, Hui Yang, Song Li

**Affiliations:** ^1^ Multidisciplinary Center for Pituitary Adenomas of Chongqing, Department of Neurosurgery, Xinqiao Hospital, Army Medical University, Chongqing, China; ^2^ Department of Neurosurgery, People’s Hospital of Shapingba District, Chongqing, China; ^3^ Department of Histology and Embryology, Chongqing Medical University, Chongqing, China; ^4^ Chongqing Institute of Brian and Intelligence, Guangyang Bay Laboratory, Chongqing, China

**Keywords:** growth hormone-secreting pituitary adenomas, DRP1, mitochondrial fission, stat3, invasion, proliferation

## Abstract

The invasiveness and high proliferation rate of growth hormone-secreting pituitary adenomas (GHPAs) are closely related to poor prognosis in patients. We previously reported that abnormal glycolysis participates in this process; however, the role of mitochondria in the invasion and proliferation of GHPAs remains unknown. In the current study, stereological methods were first used to quantitatively calculate the number and morphology of mitochondria. The results revealed that the numbers, volumes and membrane areas of mitochondria were decreased in invasive GHPAs (IGHPAs) samples compared to noninvasive GHPAs (NIGHPAs) samples. Furthermore, significantly downregulated mRNA and protein levels of dynamin-related protein 1 (Drp1) were detected in IGHPAs, but no notable changes in fusion related molecules (Mfn1, Mfn2 and OPA1) were detected, suggesting that the abnormal mitochondrial dynamics in IGHPAs are characterized by hypofission. Mitochondrial hypofission caused by Mdivi-1, a specific Drp1 inhibitor, enhanced the invasion and proliferation of GH3 cell lines and primary cells from patients with GHPAs *in vitro* and *in vivo*, while overexpression of Drp1 reversed these processes. Mechanistically, mitochondrial hypofission might activate signal transducer and activator of transcription 3 (STAT3). Specifically, elevated nuclear pSTAT3^Y705^ may promote GH3 cell invasion by upregulating the activity of matrix metalloproteinase 2/9, and elevated mitochondrial pSTAT3^S727^ may promote GH3 cell proliferation by inhibiting the mitochondria-dependent apoptotic pathway. Taken together, our findings suggest that mitochondrial hypofission induced by Drp1 might strengthen the invasion and proliferation of GHPA tumor cells by activating STAT3, providing us with a new perspective on how mitochondria regulate the development of IGHPAs.

## Introduction

Growth hormone-secreting pituitary adenomas(GHPAs) are a common subtype of pituitary adenomas (PAs) that can cause neurological dysfunctions induced by the tumor mass effect, as well as endocrine symptoms induced by growth hormone (GH) hypersecretion, and increase the mortality of patients by approximate 2-fold (1). Surgery is regarded as the first-line treatment for GHPAs, and results in an initial endocrine remission rate of 80% for microadenomas and 50% for macroadenomas (1, 2).However, the remission rate for tumors invading the cavernous sinus drops to 35%, and five-year disease recurrence rates range from 2 to 8% (1, 2). Moreover, postsurgical pathological factors (e.g., Ki-67 index, sparsely granulated adenoma) are also correlated with the prognosis of GHPAs (1, 3). A multicenter case–control study found that invasive and highly proliferative tumors presented with an increased probability of tumor persistence or progression of 25- or 12-fold, respectively (4). Therefore, intensive study on invasive and highly proliferative GHPAs is essential for the development of new therapies.

Metabolic reprogramming is well recognized as a hallmark of cancer and plays important roles in tumorigenesis (5). Transcriptiomics data (6) and metabolomics investigations (7) supported the potential oncogenetic roles of metabolic dysregulation in PAs. Our group has reported that dysregulated glucose metabolism (8), cholesterol metabolism (9) and glutamine metabolism (10) might play important roles in the tumorigenesis of PAs. For example, we have reported that lactate dehydrogenase A (LDHA) enhances the glycolysis in pituitary GH3 cells, and then promotes the invasion and proliferation of GH3 cells (8). Recently, Zhang et al. further illustrated that overproduction of lactate promoted the invasion of tumor cells *via* M2-like macrophage polarization (11). Beyond abnormal glycolysis in cancer, mitochondrial dysfunction associated with energy metabolic reprogramming has also been confirmed (12).Several tricarboxylic acid cycle intermediates (e.g., 2-hydroxyglutarate, succinate and fumarate) induced by mutant metabolic enzymes in mitochondria are well recognized as oncometabolites (13–15). Oncocytoma, a special subset of pituitary adenoma, is characterized by mitochondrial hyperplasia. Kurelac reported that mitochondrial DNA (mtDNA) mutation might disrupt respiratory complex I and induce the oncocytic phenotype of pituitary adenoma (16). Feng et al. found that hyperplastic mitochondria were characterized by metabolic changes as a result of respiratory complex I dysfunction and inefficient oxidative phosphorylation in oncocytoma (17). However, whether mitochondrial dysfunction participates in the development of GHPAs is still unclear.

Mitochondrial succinate dehydrogenase (SDH) mutations were identified in familial patients with GHPAs. In animal models with *Sdh^+/-^* mice, the adenohypophysis presented dramatic hyperplasia, and pituitary cells displayed morphological abnormalities of mitochondria and high expression of HIF1a, which suggested the role of mitochondrial metabolic enzymes in the development of GHPAs (18). Mitochondrial function is not only affected by mitochondrial metabolic enzymes, but also determined by mitochondrial morphology to some extent (19). Mitochondrial morphology is highly dynamic between fission and fusion cycles, referred to as mitochondrial dynamics. Mitochondrial dynamics are crucial for cellular processes, such as apoptosis, cell cycle and cell death (19). Several studies have indicated that mitochondrial dynamics might regulate tumor growth and metastasis (20, 21). It has been reported that the underlying mechanism of dopamine agonists in treating GHPAs might activate the mitochondrial apoptosis pathway (22). These data provide instructive clues on the role of mitochondria in GHPAs. However, whether mitochondrial dynamics regulate the aggressive behavior and proliferation of GHPAs remains largely unknown.

In the current study, we investigated mitochondrial morphology and number in invasive GHPAs (IGHPAs) samples, and then explored the underlying mechanisms by which mitochondrial dynamics regulate the invasion and proliferation of GH3 cell lines and human primary GHPAs cells.

## Materials and Methods

### Patient Selection

In this study, a total of 42 samples were obtained from patients with GHPAs, who underwent surgery in our department. IGHPAs and NIGHPAs samples accounted for 50% each. Among them, 14 GHPAs (7 IGHPAs, 7 NIGHPAs) were used for stereological study. Diagnoses of individual tumors were based on clinical signs, endocrine evaluation and postoperative pathological results. Tumor invasiveness was determined according to the Knosp classification combined with intraoperative findings (4). Grade III – IV tumors were defined as invasive tumors, and grade 0 - II tumors were noninvasive tumors. This study followed the Helsinki declaration and the supervision of the ethics committee of the Army Medical University. All patients provided written informed consent to participate in this study.

### Electron Microscopic Observation and Stereology

Electron microscopic sectioning was performed at the Center for Biological Analysis and Testing at Army Medical University. A total of 2 slices were extracted for each case by equal distance at random. Twenty-five images were acquired from each slice magnified to 25 Kd using a Japanese projection electron microscope GEM 1400 plus in accordance with X, Y axis interval of 10 μm each. From these images, stereological analyses were performed by using the Standard Program Image Analysis System of the OLYMPUS microscope (23). The mitochondrial volume fraction(Vv) was obtained by stereological point measurement.


Vv=P/P'/V

P is the number of points to hit the mitochondria. P’ is the total number of measuring points. V is the tumor volume measured by enhanced magnetic resonance imaging. The density of mitochondrial surface area(Sv) was obtained using the linear intersection technique.


Sv=2I/L'/V

I is the number of intersections between the lines and the boundary of the mitochondria. L is the total length of the line. The density of mitochondrial number (Nv) was obtained by means of the stereological box technique.


Nv=N/(S×H)/V

N is the number of mitochondria in the stereological box by the forbidden line rule. S is the area of the forbidden line frame. H is the height of the forbidden line frame.

### Reverse Transcription and Real-Time Quantitative PCR Technique

Total RNA of GHPAs was extracted using TRIzol (Toyobo, Osaka, Japan) and reverse transcribed into cDNA utilizing PrimeScript^®^ RTase (Toyobo, Osaka, Japan). cDNA was amplified using SYBR premix Taq TM II (Toyobo, Osaka, Japan) and CFX96 real-time (Bio-Rad Laboratories, Hercules, CA, USA). The relative transcription level of the gene was calculated using the 2−^△△^ct method. The primer sequences (5’-3’) used for qPCR were as follows: Mfn1 F-GTGGCAAACAAAGTTTCATGTG, R-CACTAAGGCGTTTACTTCATCG; Mfn2 F- CTCTCGCAGAAGGCTTTCAAGT, R-TTCACGCATTTCCTCGCAGTA; OPA1 F- TCTGCACACTCAGTTGAAGTAT, R-GCCTTTGTCATCTTTCTGCAAT; Drp1 F- CATGAGACTTTTGGGCGAACC, R-GGCACAAATAAAGCAGGACGAG; β-actin F-GCACCACACCTTCTACAATGAGC, R-TAGCACAGCCTGGATAGCAACG.

### Immunohistochemistry

GHPAs tissues were fixed in paraformaldehyde for 24 hours; and then embedded in paraffin. Paraffin sections were sliced at a thickness of 5 µm for subsequent immunohistochemical staining. Staining of sections was performed in accordance with the standard procedures described in our previous studies. The sections were incubated with anti-Drp1 (1:200; ab56788, Abcam, Cambridge, UK), anti-STAT3 (1:200; ab68153, Abcam), anti-phospho-STAT3 (S727) (1:150; ab32143, Abcam), and anti-phospho-STAT3 (Tyr705) (1:150; #9145, Cell Signaling Technology, Danvers, USA) primary antibodies overnight at 4°C. The sections were then incubated with secondary immunoglobulin conjugated to peroxidase-labeled dextran polymer for 1 h at 37°C. 3,3-Diaminobenzidine (Boster, Wuhan, China) was applied to view the immunoreactions. Finally, these slices were stained with hematoxylin, dehydrated, and covered with coverslips. Images of different sections were obtained by fluorescence microscopy (TSC-TIV; Leica, Nussloch, Germany). Negative control experiments lacked the primary antibodies.

### Cell Lines and Primary Cells

Rat GH3 pituitary adenoma cell lines were purchased from the American Type Collection (ATCC, Manassas, VA, culture, USA). Cells were cultured in Ham’s F-12K media containing 2.5% fetal bovine serum and 15% horse serum and placed in a humidified incubator with a 5% CO_2_-humidified atmosphere at 37°C. Five primary GHPA cells were obtained from patients who were surgically treated at Xinqiao Hospital. The primary cells were maintained in 10% FBS–containing MEM and cultured in a 5% CO_2_-humidified atmosphere at 37°C.

### Lentivirus and Transfection

Lentiviral vectors (OBIO, Shanghai, China), including empty vector and Drp1 overexpression vector, were transfected into GH3 cells at a multiplicity of infection (MOI) of 100 according to the manufacturer’s instructions. Stable colonies were identified by intense mCherry fluorescence. The upregulation efficacy of Drp1 protein was verified by western blotting.

### Mitochondrial Division Inhibitor and STAT3 Inhibitor

Mdivi-1 (338967-87-6, Selleck, Shanghai, China), a mitochondrial division inhibitor, is a highly efficient small molecule that selectively inhibits the activity of Drp 1 GTPase by blocking the self-assembly of Drp1 (24). Mdivi-1 was formulated as 10 mM liquid storage with dimethylsulfoxide, and was used to inhibit Drp1 at a concentration of 10 μM. HO-3867(HY-100453, MCE, USA), an analog of curcumin, is a selective STAT3 inhibitor that inhibits STAT3 phosphorylation, transcription, and DNA binding activity without affecting the expression of other active STATs. HO-3867 was formulated as 10 mM liquid storage with dimethylsulfoxide, and was used to inhibit STAT3 at a concentration of 10 μM (25). Cryptotanshinone(Cry) (HY-N0174, MCE) is a STAT3 inhibitor, that strongly inhibits the phosphorylation of STAT3 Tyr705, and has a weak effect on STAT3 Ser727. Cry was formulated as 10 mM liquid storage with dimethylsulfoxide, and was used to inhibit STAT3 at a concentration of 7 μM (26).

### Protein Extracts and Western Blotting

Total proteins were extracted from GHPAs tissue or GH3 cells using total protein extraction kits (Bestbio, Shanghai, China). Mitochondrial proteins and nuclear proteins were extracted with kits (Beyotime Biotech, Shanghai, China). Extracts equivalent to 50 µg of protein were integrated into SDS-PAGE gels and then transferred onto polyvinylidene difluoride membranes. The membranes were blocked in 5% nonfat milk with Tris-buffered saline containing 0.05% Tween 20 for 3 hours at room temperature and incubated with mouse antibodies against Drp1 (1:200; ab56788, Abcam) and β-Actin (1:1000; ab8227, Abcam) and with rabbit antibodies against Phospho-Drp1 (Ser616)(1:1000; #4494, Cell Signaling Technology), Phospho-Drp1 (Ser637)(1:1000; ab193216, Abcam), STAT3 (1:300; ab68153, Abcam), STAT3 (phospho S727) (1:250; ab32143, Abcam), STAT3 (phospho Tyr705) (1:250; #9145, Cell Signaling Technology), Bcl-2 (1:1000; ab59348, Abcam), Bax (1:1000; ab32503, Abcam), and Caspase 3 (1:500; #9662, Cell Signaling Technology). Then, the membranes were further incubated with horseradish peroxidase-conjugated goat anti-mouse (1:2000, ZB-2305, ZSGB-BIO, Beijing, China) and anti-rabbit (1:2000; sc-2012, Santa Cruz Biotechnology) IgG secondary antibodies. The membrane signals were visualized using a gel imaging chemiluminescence system (Fluor Chem, ProteinSimp, USA).

### Cell Counting Experiment

The number of cells was quantified using the WST-8 Cell Counting Kit-8 (Dojindo Laboratories, Mashiki-machi, Kumamoto, Japan) in accordance with the manufacturer’s instructions. A total of 2.0*10^5^ cells were initially cultured in 96-well plates and harvested after drug intervention for 48 hours. Cell numbers were quantified according to the manufacturer’s instructions.

### Cell Invasion Assay

Cells were starved in serum-free medium for 12 hours. Then, cells (GH3, 5×10^5^/well, primary tumor cells, 3×10^5^) were suspended in 150 µl serum-free medium and placed into the upper chamber (8-mm pore; Costar, Bethesda, MD, USA) that had been precoated with 70 μl Matrigel at a concentration of 300 μg/ml (BD Biosciences, USA). Five hundred microliters of whole serum medium containing 2.5% FBS and 15% HS was added to the lower chamber. After incubation with the indicated treatments for 24 hours, the culture medium was removed, and the matrix adhesive on the bottom of the chamber was gently removed with cotton swabs. Next, the cells on the membrane were fixed in 5% paraformaldehyde for 15 min and stained with crystal violet solution (Boster). The cells on the membrane were imaged under an optical microscope (Leica, DMI3000 Bat) at a magnification of 10*20 for 10 images obtained according to random equidistant extraction.

### Detection of Matrix Metalloproteinase 2/9 (MMP2/9)

MMP2/9 enzyme activities were detected using a cell MMP2/9 *in situ* zymography fluorescence staining kit (GMS80062.2, GenMed Scientifics Inc. USA). Then, the fluorescence intensity was observed under a fluorescence microscope (BX63, Olympus, Japan) and analyzed by Image-Pro Plus 6.0.

### Cell Cycle Analysis

In total, 2.0×10^6^ cells in each group were harvested and used for cell cycle analysis. The cells were washed with PBS three times and then incubated in 70% alcohol at 4°C overnight. Subsequently, the cells were stained in 0.05 mg/ml propidium iodide (PI; BD Biosciences Pharmingen) and analyzed by flow cytometry (FACScan; BD Biosciences Pharmingen, San Diego, CA, USA). Cell debris, cell doublets, and cell clumps were excluded from the analysis. DNA histograms were created using ModFit LT V2.0 software.

### Apoptosis Analysis

In total, 2.0*10^6^ cells in each group were harvested and used for apoptosis analysis. Apoptosis was assessed using a FITC-Annexin V apoptosis detection kit (556547, BD Biosciences Pharmingen) according to the manufacturer’s instructions. Cells were stained with FITC-Annexin V and PI. Apoptosis was detected by flow cytometry and analyzed further using ModFit LT V2.0 software.

### Analysis of Mitochondrial Membrane Potential and Reactive Oxygen Species (ROS)

Approximately 1×10^6^ GH3 cells were collected for mitochondrial membrane potential and ROS detection using flow cytometry. Mitochondrial membrane potential was detected by rhodamine 123 using a mitochondrial membrane potential detection kit (C2008S, Beyotime Biotech), and ROS was determined by a DCFH-DA reactive oxygen species assay kit (S0033S, Beyotime Biotech) according to the manufacturer’s recommendations. Each sample was assessed by flow cytometry for fluorescence intensity. The results were analyzed by ModFit LT V2.0 software.

### *In Vivo* Experiments

*In vivo* xenograft experiments were performed similar to our previous study (9). Twenty-eight 4 weeks old male BALB/cA-nu mice were purchased from Charles River (Beijing, China) and housed under SPF conditions. Then, the animals were randomly divided into 4 groups (7 mice/group). A total of 5×10^6^ transfected GH3 cells suspended in 100 µl of solution (50% PBS and 50% Matrigel) were subcutaneously inoculated into the right flank of the mice. Treatment with Mdivi-1 was started 2 weeks after inoculation of the cells. The Mdivi-1-treated groups (VE+Mdivi-1, Drp1^++/++^+Mdivi-1) received daily intraperitoneal injection of 50 mg/kg Mdivi-1 for the next 3 weeks until sacrifice, while the other two groups (VE, Drp1^++/++^) received daily intraperitoneal injection of an equal volume of PBS only. The mice were monitored daily for any discomfort. The mice were weighed, and tumor volumes were also measured every three days. Tumor tissue was removed from tumor-bearing mice following the final treatment. Tumor volumes were calculated using the following formula: V (mm^3^) = [AB^2^]/2, where A is the tumor length and B is the tumor width. The excised tumors were weighted. All animal procedures were conducted according to protocols approved by the Institutional Animal Care and Ethics Committee.

### Statistical Analysis

Data are expressed as the means ± SEM. A two-tailed Student’s t-test was applied to determine statistical significance between the two groups. These analyses were performed using SPSS for Windows, version 18.0 (SPSS Inc., USA).

## Results

### Mitochondrial Dynamics Were Dysregulated in IGHPAs

**Figure 1:**
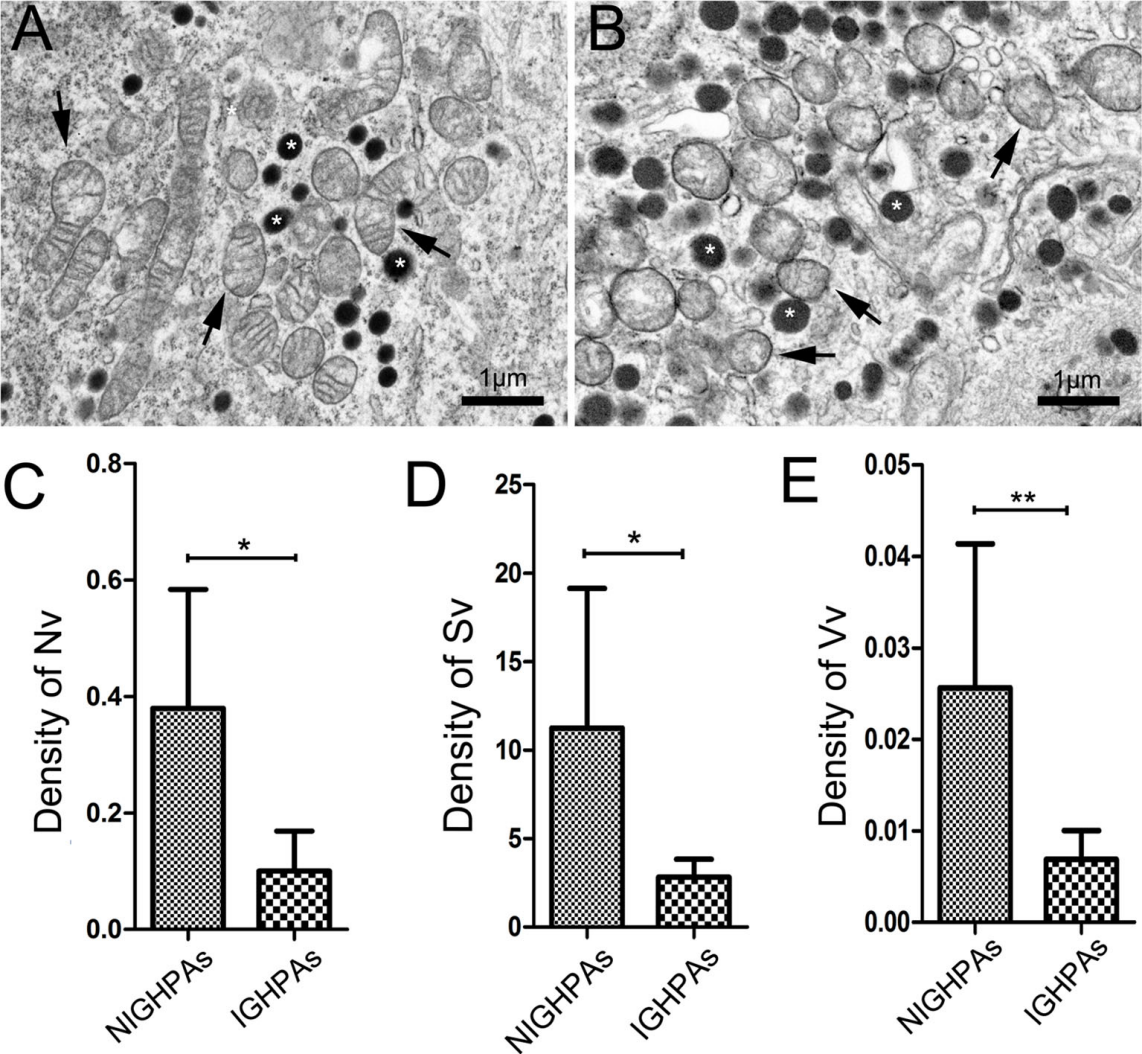
Stereological analysis of mitochondria in NIGHPAs (n = 7) and IGHPAs (n = 7). The number density, bulk density and surface area density were corrected with tumor volume measured by nuclear magnetic resonance. Nonparametric tests were used for statistical analysis. **(A)** Representative electron microscopic image of tumor cells in NIGHPAs. Several mitochondria with normal morphology (arrows) and secretory granule (stars) were observed. **(B)** Representative electron microscopic image of tumor cells in IGHPAs. Several swollen mitochondria (arrows) and secretory granule (stars) were observed. **(C)** The density of mitochondrial number (Nv) in IGHPAs was significantly lower than that of NIGHPAs. **(D)** The density of mitochondrial surface area (Sv) in IGHPAs was significantly lower than that of NIGHPAs. **(E)** The mitochondrial volume fraction (Vv) in IGHPAs was significantly lower than that of NIGHPAs. Scale bar = 1um, *P < 0.05, **P < 0.01.

The morphology and number of mitochondria were visually observed under an electron microscope and contrasted objectively by stereological measurement, including contour counting, point counting and intersection counting. Several mitochondria with normal morphology and secretory granules were observed in surgical noninvasive GHPAs (NIGHPAs) samples (**Figure 1A**). However, several swollen mitochondria were observed in IGHPAs samples (**Figure 1B**). Stereological analyses revealed that the density of mitochondrial number (Nv) and mitochondrial surface area (Sv) in IGHPAs were significantly lower than those in NIGHPAs (**Figures 1C, D**). Meanwhile, the mitochondrial volume fraction (Vv) in IGHPAs was significantly lower than that in NIGHPAs (**Figure 1E**). These stereological results suggest that mitochondrial fission and fusion might be dysregulated in IGHPAs.

### Downregulation of Drp1 Induced Mitochondrial Hypofission Promotes the Invasion of GHPAs

**Figure 2:**
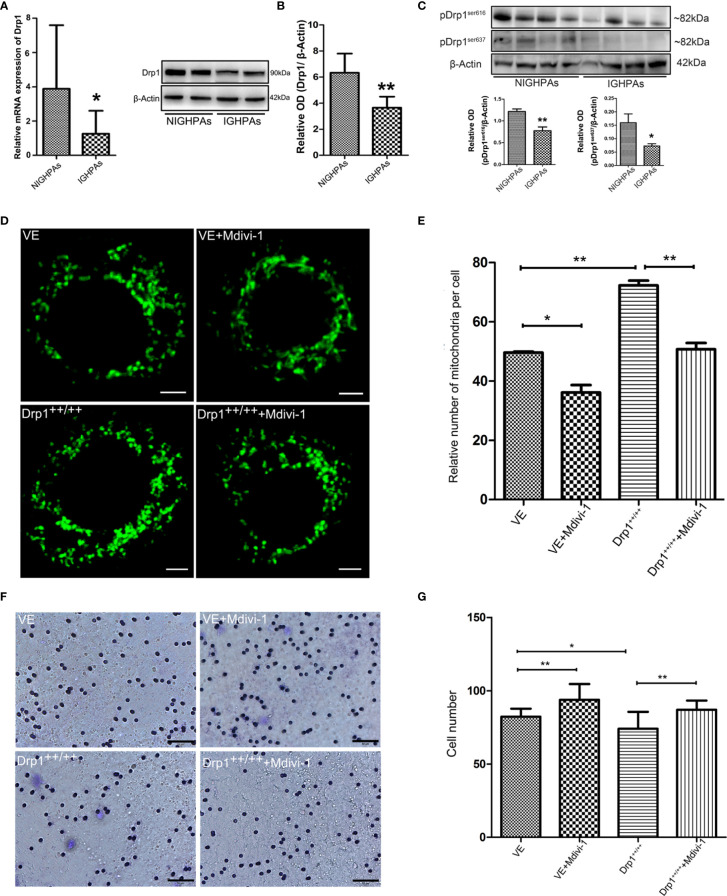
The relationship between Drp1 regulated mitochondrial dynamics and tumor invasion. **(A)** Expression of Drp1 mRNA was assessed by RT-qPCR in NIGHPAs (n = 12) and IGHPAs (n = 13) samples. **(B)** Expression of Drp1 protein levels in NIGHPAs (n =8) and IGHPAs (n =8) samples was assessed by western blotting (left panel). Statistical analysis of the western blotting results (right panel). **(C)** Protein levels of phosphorylated Drp1at Ser616 and Ser637 in NIGHPAs and IGHPAs samples were assessed by western blotting (above panel). Statistical analysis of the western blotting results (below panel). **(D)** Mitochondria were stained with Mito-Tracker Green FM to display quantity and mitochondrial morphology in four GH3 cell lines groups, VE (transfected with empty vector) group, VE+Mdivi-1 group, Drp1^++/++^(Drp1 over expression vector) group, Drp1^++/++^+Mdivi-1 group. GH3 cells were observed after intervention with Mdivi-1 for 48 hours by confocal microscope. Scale bar = 2.5 um. **(E)** Statistical analysis of the relative number of mitochondria per cell (n = 25). **(F)** The invasive ability of GH3 cells was evaluated by transwell assay (n = 3). Scale bar = 50 um. **(G)** Statistical analysis of the invasive GH3 cell number. *P < 0.05, **P < 0.01.

The morphology and number of mitochondria are highly dynamic and determined by the processes of fusion and fission, which are affected by mitochondrial dynamics proteins, mainly mitofusin (Mfn), optic atrophy protein 1 (OPA1) and dynamin-related protein 1 (Drp1) (19). We detected the mRNA expression levels of the mitochondrial fusion related proteins (Mfn1, Mfn2 and OPA1), and observed no significant difference between NIGHPAs and IGHPAs samples (**Figures S1A–C**). However, the mRNA expression levels of the mitochondrial fission related protein Drp1 were significantly downregulated in IGHPAs samples (**Figure 2A**). Further western blotting experiments confirmed that the protein levels of Drp1 were also significantly downregulated in IGHPAs samples (**Figure 2B**). Accordingly, two main phosphorylation sites (Ser616 and Ser637) of Drp1 protein were detected significantly downregulated in IGHPAs samples (**Figure 2C**). These results indicate that the dysregulated mitochondrial dynamics confirmed by stereological measurement in IGHPAs might be a result of Drp1 induced mitochondrial hypofission. Furthermore, we verified the effect of Drp1 protein on mitochondrial fission and tumor cell invasion using GH3 cell lines *in vitro*. We successfully constructed a Drp1 overexpression (Drp1^++/++^) GH3 cell model (data not shown) by lentivirus transfection and used Mdivi-1 to specifically inhibit Drp1 (24). The number of mitochondria was significantly increased in response to overexpression of Drp1 in GH3 cells but decreased significantly in response to Mdivi-1 (**Figures 2D, E**), which were further confirmed by transmission electron microscopy (**Figure S1D**). Then, we evaluated whether inhibition of Drp1 might affect GH3 cell invasion. Transwell experiments demonstrated that overexpression of Drp1 attenuated the invasion of GH3 cells, while inhibition of Drp1 by Mdivi-1 promoted cell invasion (**Figures 2F, G**).

### Inhibition of Drp1 Promotes GH3 Cells Invasion by Activating STAT3

**Figure 3:**
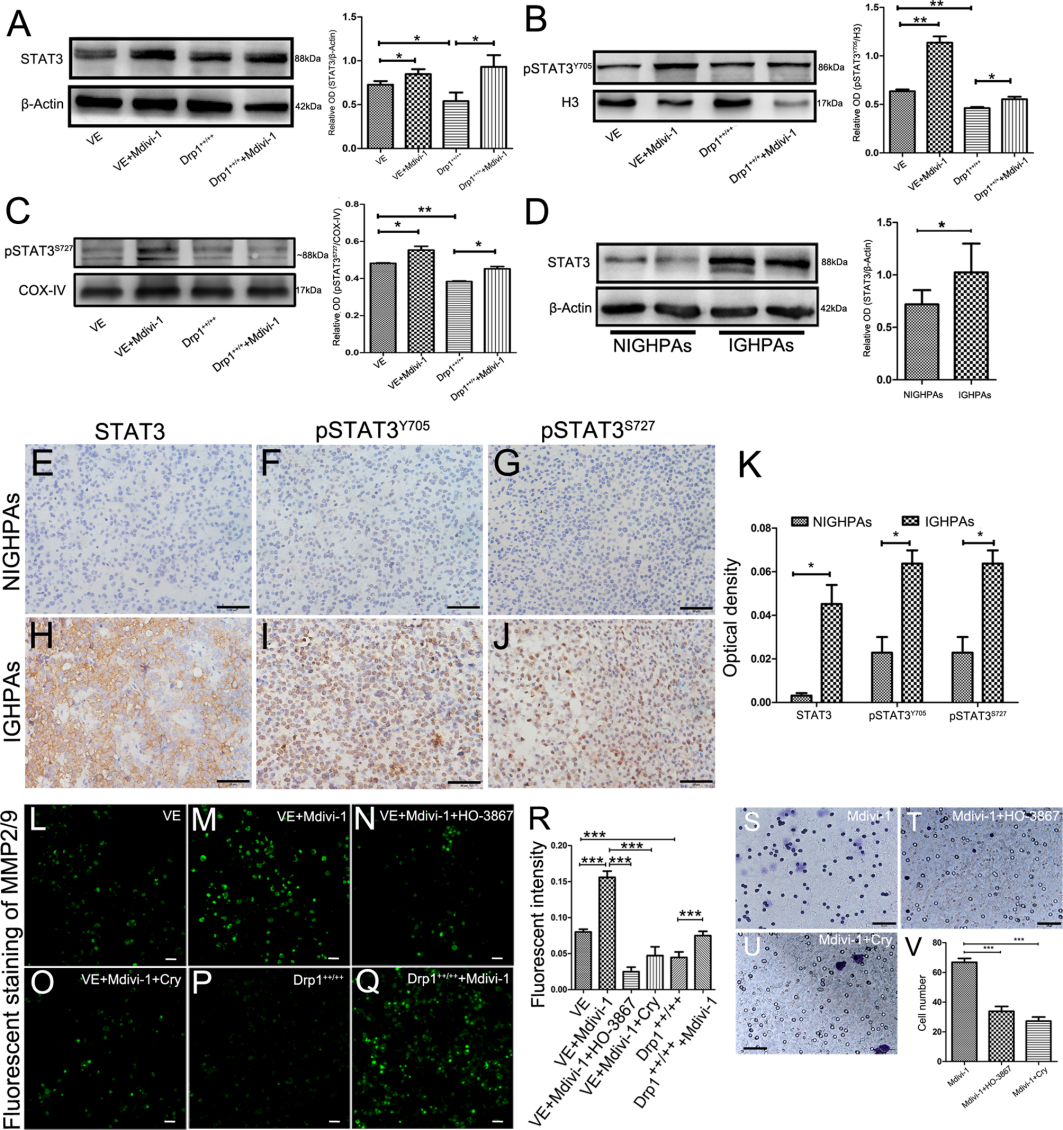
Inhibition of Drp1 enhanced the invasion of GH3 cells *via* activating STAT3. **(A)** The expression of STAT3 protein was detected by western blotting in four GH3 cell groups (VE, VE+Mdivi-1, Drp1^++/++^, Drp1^++/++^+Mdivi-1) after 48 hours (n = 3) (left panel). Statistical analysis of the western blotting results (right panel). **(B)** Nuclear pSTAT3^Y705^, a phosphorylated form of STAT3 and mainly involved in nuclear transcription, was detected by western blotting in the four groups. H3 was used as a reference (n = 3) (left panel). Statistical analysis of the western blotting results (right panel). **(C)** Mitochondrial p-STAT3^S727^, another phosphorylated form of STAT3 and mainly distribute in mitochondria, was detected by western blotting in the four groups. COX-IV was used as a reference (n = 3) (left panel). Statistical analysis of the western blotting results (right panel). **(D)** Expression of STAT3 protein levels in NIGHPAs (n =8) and IGHPAs (n = 8) samples were assessed by western blotting (left panel). Statistical analysis of the western blotting results (right panel). **(E–G)** IHC staining of STAT3, pSTAT3^Y705^, pSTAT3^S727^ in NIGHPAs samples (n = 10). **(H–J)** IHC staining of STAT3, pSTAT3^Y705^, pSTAT3^S727^ in IGHPAs samples (n = 10). **(K)** Statistical analysis of the IHC results. **(L–Q)** Activity of MMP2/9 were detected by situ zymography fluorescence staining in six groups (VE, VE+Mdivi-1, VE+Mdivi-1+HO-3867, VE+Mdivi-1+Cry, Drp1^++/++^, Drp1^++/++^+Mdivi-1). **(R)** Statistical analysis of the MMP2/9 fluorescent intensity. **(S–U)** GH3 cell invasion were evaluated by transwell assay when treated with Mdivi-1, Mdivi-1+HO-3867, Mdivi-1+Cry. **(V)** Statistical analysis of the invasive GH3 cell number in the three groups. HO-3867, a broad spectrum inhibitor of STAT3 phosphorylation. Cry, a selective inhibitor of STAT3 phosphorylation at Tyr705. **(E–J, S-U)**, Scale bar =50 μm. L-Q, Scale bar =25 μm. *P < 0.05, **P < 0.01, ***P < 0.001. Data were expressed as mean ± SEM.

The mitochondrial membrane has been well recognized as a platform that mediates the transduction of signals into and out of the mitochondria, which supports the role of mitochondrial dynamics in regulating cell signaling pathways (19). It is well known that the signal transducer and activator of transcription 3 (STAT3) signaling pathway is involved in cellular proliferation, invasion and apoptosis; thus, we detected whether Drp1 affects STAT3. We found that inhibition of Drp1 by Mdivi-1 upregulated the expression of total cell STAT3 protein, and downregulated STAT3 protein in Drp1^++/++^ GH3 cells (**Figure 3A**). Moreover, the phosphorylated site of STAT3 at Y705 (pSTAT3^Y705^), an active form of STAT3 that is primarily involved in STAT3 nuclear transcription, was upregulated in the nuclei of GH3 cells when Drp1 was inhibited by Mdivi-1. Accordingly, protein levels of pSTAT3^Y705^ were downregulated in Drp1^++/++^ GH3 cells (**Figure 3B**). Another active form of STAT3 that is primarily distributed in mitochondria, STAT3 phosphorylated at S727 (pSTAT3^S727^), was also upregulated in the mitochondria of GH3 cells when Drp1 was inhibited by Mdivi-1 and downregulated in Drp1^++/++^ GH3 cells (**Figure 3C**). Similarly, we found that expression levels of STAT3 protein were significantly higher in the IGHPAs samples (**Figure 3D**). Further immunohistochemical staining confirmed the overexpression of STAT3, pSTAT3^Y705^ and pSTAT3^S727^ in the IGHPAs samples (**Figures 3E–K**). STAT3 is known to activate matrix metalloproteinase 2/9 (MMP2/9) gene expression, which could mediate an enhancement effect on tumor invasiveness (27). We analyzed the enzyme activity of MMP2/9 in GH3 cells and found that the activity of MMP2/9 was increased by inhibition of Drp1. STAT3 inhibitors significantly reversed this effect, which was similar to the effect of Drp1 overexpression (**Figures 3L–R**). To explore the role of STAT3 in GH3 cells invasion induced by Mdivi-1, transwell assays were performed. We found that STAT3 inhibitors blocked GH3 cell invasion enhanced by inhibition of mitochondrial fission (**Figures 3S–V**).

### Inhibition of Drp1 Enhances the Proliferation of GH3 Cells

**Figure 4:**
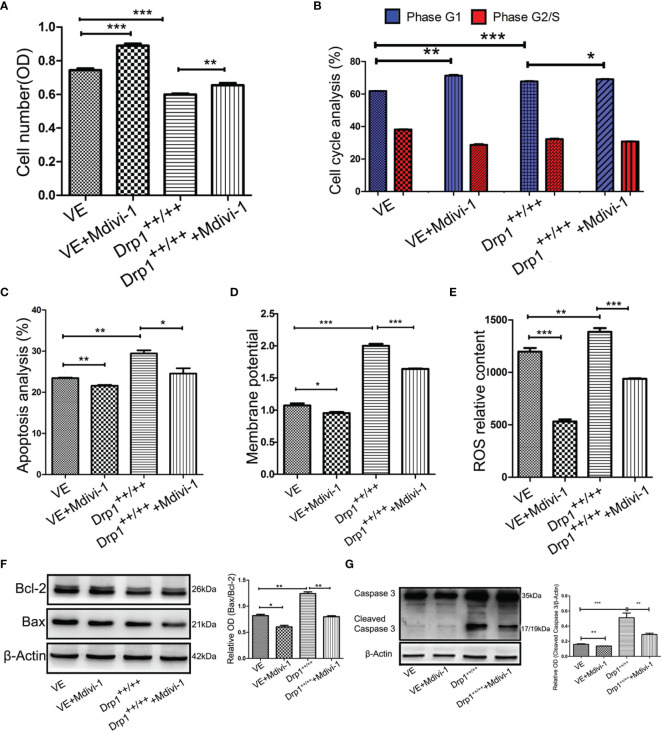
Inhibition of Drp1 promoted proliferation of GH3 cells. **(A)** The number of GH3 cells in four groups (VE, VE+Mdivi-1, Drp1^++/++^, Drp1^++/++^+Mdivi-1) at 48 h were assessed by CCK-8 assay (n = 3). **(B)** Cell cycle at 48 h were analyzed by flow cytometry (n = 3). **(C)** Cell apoptosis at 48 h were analyzed by flow cytometry (n = 3). **(D)** Mitochondrial membrane potential of four GH3 cell groups were detected by flow cytometry using Rhodamine123 at 48 h (n = 3). **(E)** Reactive oxygen species (ROS) were detected by flow cytometry using DCF-DA fluorescence at 48 h (n = 3). **(F)** The expression of Bcl-2 and Bax proteins were detected by western blotting (n = 3) (left panel). Statistical analysis of the western blotting results (right panel). **(G)** The expression of cleaved caspase-3 protein was detected by western blotting (n = 3) (left panel). Statistical analysis of the western blotting results (right panel). *P < 0.05, **P < 0.01, ***P < 0.001. Data were expressed as mean ± SEM.

To further investigate whether inhibition of Drp1 regulates GH3 proliferation, we inspected GH3 cell number by CCK-8, cell cycle and apoptosis by flow cytometry. The CCK-8 assay confirmed that inhibition of Drp1 enhanced the proliferation of GH3 cells (**Figure 4A**), which was due to promotion of the cell cycle (**Figure 4B**, **Figure S2A**) and protection from apoptosis (**Figure 4C**, **Figure S2B**). Moreover, we detected whether inhibition of Drp1 could directly affect the mitochondrial apoptosis pathway. GH3 cells treated with the Drp1 inhibitor Mdivi-1 presented reduced mitochondrial membrane potential, and Drp1^++/++^ GH3 cells presented increased mitochondrial membrane potential, which could be reversed by Mdivi-1(**Figure 4D**, **Figure S2C**). We also found that reactive oxygen species (ROS) underwent similar changes. (**Figure 4E** and **Figure S2D**). Then, we measured the expression of mitochondrial apoptotic pathway-related proteins, including Bcl-2, Bax and cleaved caspase-3. Western blotting results indicated that the proapoptotic protein levels of Bax/Bcl-2 (**Figure 4F**) and cleaved caspase-3 (**Figure 4G**) were significantly decreased by the Drp1 inhibitor Mdivi-1.

### Drp1 May Regulate the Proliferation of GH3 Cells *Via* Mitochondrial STAT3 Signaling

**Figure 5:**
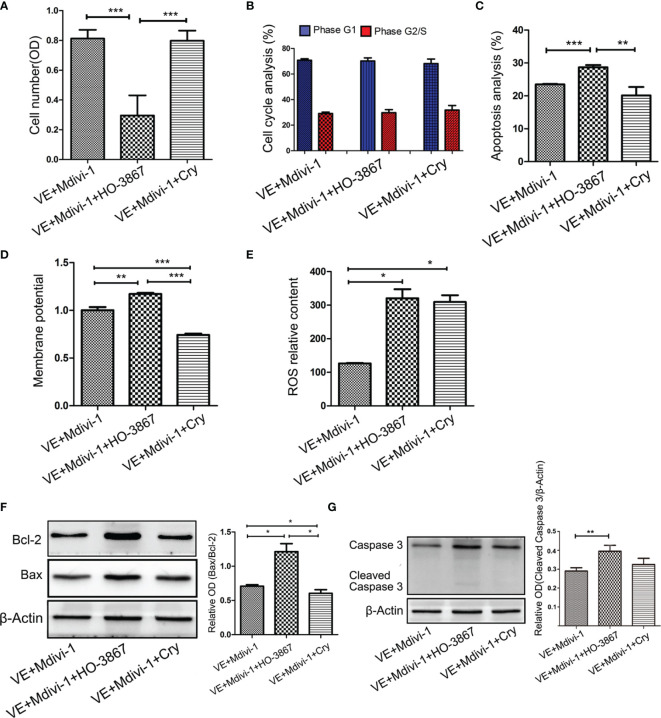
Inhibition of STAT3 reversed the Drp1 regulating pro-proliferation of GH3 cells. **(A)** The number of GH3 cells in three groups (VE+Mdivi-1, VE+Mdivi-1+HO-3867, VE+Mdivi-1+Cry) at 48 h were assessed by CCK-8 assay (n = 3). **(B)** Cell cycle at 48 h were analyzed by flow cytometry (n = 3). **(C)** Cell apoptosis at 48 h were analyzed by flow cytometry (n = 3). **(D)** Mitochondrial membrane potential of three GH3 cell groups were detected by flow cytometry using Rhodamine123 at 48 h (n = 3). **(E)** Reactive oxygen species (ROS) were detected by flow cytometry using DCF-DA fluorescence at 48 h (n = 3). **(F)** The expression of Bcl-2 and Bax proteins were detected by western blotting (n = 3) (left panel). Statistical analysis of the western blotting results (right panel). **(G)** The expression of cleaved caspase-3 protein was detected by western blotting (n = 3) (left panel). Statistical analysis of the western blotting results (right panel). *P < 0.05, **P < 0.01, ***P < 0.001. Data were expressed as mean ± SEM.

To further investigate whether the STAT3 signaling pathway is involved in the process through which Drp1 regulates GH3 cell proliferation, we chose two STAT3 inhibitors, HO-3867 (a broad spectrum inhibitor of STAT3 phosphorylation) and Cry (a selective inhibitor of STAT3 phosphorylation at Tyr705), for *in vitro* experiments. The CCK-8 assay indicated that HO-3867 sufficiently reversed the proliferation of GH3 cells induced by Mdivi-1, but Cry had no effect (**Figure 5A**). Neither inhibitor affected the cell cycle (**Figure 5B** and **Figure S3A**). We further observed a higher rate of apoptotic cells treated with VE+Mdivi-1+HO-3867 while Cry had no effect compared to VE+Mdivi-1 (**Figure 5C** and **Figure S3B**). Moreover, HO-3867 significantly increased the mitochondrial membrane potential and ROS (**Figures 5D, E** and **Figures S3C, D**). Accordingly, western blotting results indicated that the proapoptotic protein levels of Bax/Bcl-2 (**Figure 5F**) and cleaved caspase-3 (**Figure 5G**) were significantly upregulated by HO-3867 but not Cry.

### Inhibition of Drp1 Promotes the Invasion and Proliferation of MMQ Cell Lines and Human PAPCs

We further investigated whether inhibition of Drp1 affects the invasion and proliferation of MMQ cell lines and human primary GHPAs cells (PAPCs). Similar to GH3 cells, the Drp1 inhibitor Mdivi-1 significantly enhanced the invasion of MMQ cells (**Figures S4A, B**) and PAPCs (**Figures S4D, E**), and this effect was reversed by STAT3 inhibitors HO-3867 and Cry. The CCK-8 assay further confirmed that the Drp1 inhibitor Mdivi-1 significantly promoted the proliferation of MMQ cells (**Figure S4C**) and PAPCs (**Figure S4F**), and this pro-proliferative effect was reversed by the STAT3 inhibitor HO-3867.

### Inhibition of Drp1 Promotes the Growth and Invasion of GH3 Cells *In Vivo*

**Figure 6:**
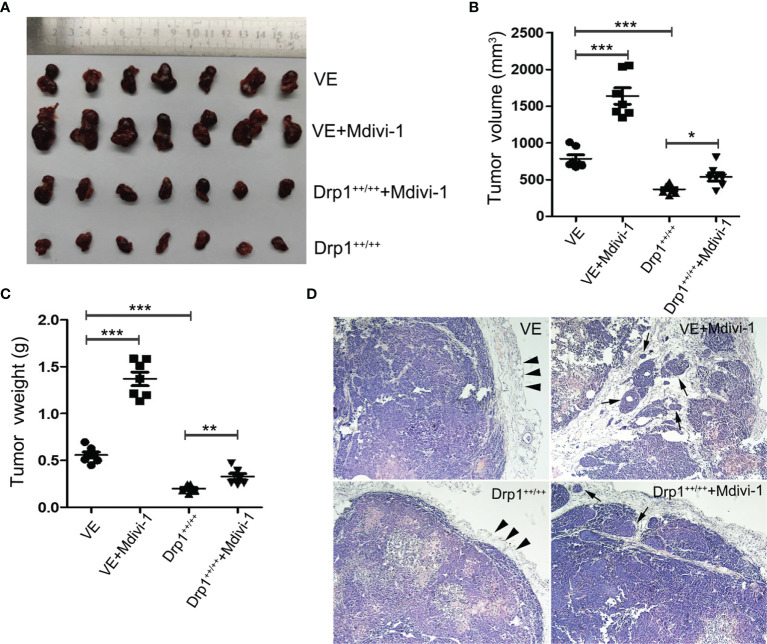
Inhibition of Drp1 promoted the growth and invasion of GH3 cells *in vivo*. **(A)** Excised tumors in four groups (VE, VE+Mdivi-1, Drp1^++/++^, Drp1^++/++^+Mdivi-1) were shown. **(B)** Tumor volume in different groups. **(C)** Tumor weight in different groups. **(D)** HE staining of tumor(×100 magnification). Smooth tumor capsule (arrowheads) was detected in VE and Drp1^++/++^ group. Tumor nodules (arrows) were detected in tumor capsule in VE+Mdivi-1 and Drp1^++/++^+Mdivi-1 group. *P < 0.05, **P < 0.01, ***P < 0.001.

To further investigate the effects of Drp1 on PA cells *in vivo*, a PA xenograft model was generated by subcutaneous injection of either vector control (VE) or Drp1^++/++^ GH3 cells into nude mice. The mice were randomly divided into four groups (VE, VE+Mdivi-1, Drp1^++/++^, DRP1^++/++^+Mdivi-1). We found that inhibition of Drp1 significantly promoted the growth of GH3 cells *in vivo*, and overexpression of Drp1 attenuated the tumor growth (**Figures 6A–C**). Further, HE staining confirmed that several tumor nodules were detected in tumor capsule in VE+Mdivi-1 group, but not in VE and Drp1^++/++^ group, which indicated that inhibition of Drp1 enhanced the tumor invasion (**Figure 6D**).

## Discussion

Increasing evidence suggests that abnormal mitochondrial dynamics participate in the processes of tumorigenesis (28, 29). In the current study, stereological results confirmed dysregulated mitochondrial dynamics of IGHPAs. Furthermore, we identified that downregulation of Drp1 was responsible for the inhibition of mitochondrial fission and might be related to the invasion and proliferation of IGHPAs. Inhibition of mitochondrial fusion enhanced the invasion and proliferation of GH3 cell lines and human primary GH-PA cells *in vitro* and *in vivo*, and overexpression of Drp1 reversed this process. Mechanistically, decreased mitochondrial fission inhibited mitochondrial pathway-mediated apoptosis and upregulated the activity of MMP2/9 in GH3 cells *via* activation of the transcription factor STAT3.

Despite supplying energy, mitochondria play a key role in tumor progression by regulating redox homeostasis, oncogenic signaling, innate immunity, and apoptosis of cancer cells (12). Mutations in *SDH* were detected in patients with GHPAs, and further animal models confirmed that *SDH* deficiency might contribute to tumorigenesis (18, 30). Mitochondrial function is affected not only by mitochondrial metabolism, but also by mitochondrial dynamics regulating mitochondrial morphology (19). In the current study, we found that the number, volume and membrane area of mitochondria were decreased in IGHPAs using stereological methods. Downregulation of mitochondrial membrane area referred to decreased mitochondrial ATP through oxidative phosphorylation (28), which seems consistent with our previous research on abnormal glycolysis in IGHPAs. Therefore, our stereological results first suggested a potential role of abnormal mitochondrial dynamics in the invasion of pituitary tumors.

The core mechanisms of mitochondrial membrane dynamics are fusion and fission, which are affected by mitochondrial dynamics proteins (19, 29). The mRNA expression levels of Mfn1, Mfn2 and OPA1 were similar between the IGHPAs and NIGHPAs sample, while the mRNA and protein levels of Drp1 were significantly downregulated in IGHPAs samples. These data suggest that the primary dysregulated mitochondrial dynamics event in IGHPAs might be hypofission, but not hyperfusion. Similar to our findings, Sabatino et al. observed downregulation of Drp1 protein levels and decreased mitochondrial fission during the development of estrogen induced experimental pituitary tumors *in vivo (* 31, 32). However, upregulation of Drp1 has been reported in many cancers (e.g. breast cancer, glioblastomas) and may be a potential target for cancer treatment (21, 33). These data may seem contradictory and puzzled, however, we think they indicate the different role of mitochondria in tumorigenesis and development in various tumors (28). Furthermore, we investigated the role of mitochondrial dynamics in the invasion of GH3 cell lines *in vitro* and *in vivo*. We found that overexpression of Drp1 promoted the fission of mitochondria and attenuated the invasion of tumor cells, while inhibition of mitochondrial fission by Midiv-1 enhanced invasive behavior through upregulation of MMP2/9. The role of mitochondrial dynamics has been confirmed in the migration and invasion of different subtypes of cells. In neural stem cells, miR-137 accelerates mitochondrial fission and fusion and thereby promotes neuronal differentiation and migration (34). In a KRAS mutated carcinoma model, autophagy deficiency-induced mitochondrial hyperfission attenuated the invasion of tumor cells (35). Matrix metalloproteinase family members, primarily MMP-2 and MMP-9, have been well-recognized as core molecules responsible for the invasion of pituitary adenomas by our previous studied (8, 36) and other studies (37). Taken together, our data indicate that mitochondrial hypofission might strengthen the invasiveness of IGHPAs tumor cells.

Considering that the IGHPAs generally accompanied by highly proliferative features, we further investigated whether Drp1 mediated mitochondrial hypofission affects the proliferation of tumor cells. We found that inhibition of mitochondrial fission promoted the proliferation of GH3 cell lines *in vitro* and *in vivo*, while overexpression of Drp1 reversed this pro-proliferative effect. Mechanistically, inhibition of mitochondrial fission decreased the mitochondrial membrane potential, reactive oxygen species (ROS) and internal apoptosis stimulator responses, which restrained the mitochondria-mediated apoptosis signaling pathway. Several proapoptotic (e.g., Bax and Bak) and antiapoptotic factors (e.g., Bcl-2 family members) were identified to colocalize with the fission sites of the mitochondrial outer membrane, and translocation of these factors in dysregulated mitochondrial dynamics triggers caspase-dependent apoptosis (38). In HeLa cells, Drp1 dependent mitochondrial fission might trigger intrinsic apoptosis *via* cytochrome c release (39). Mazumder et al. also reported that nonsteroidal anti-inflammatory drugs upregulated Drp1 expression and thereby promoted mitochondrial hyperfission, which resulted in apoptosis of gastric cancer cells (40). Thus, we hypothesized that inhibition of mitochondrial fission might restrain the Bax induced caspase 3 dependent apoptosis of GH3 cells. Conversely, Drp1 dependent mitochondrial hyperfission presents antiapoptotic effects and promotes the proliferation of tumor cells in pancreatic cancer (20) and glioblastoma (33). However, Zhao et al. found that regulation of mitochondrial dynamics by altering Drp1, Mfn1 and Mfn2 had no effect on the breast cancer cell cycle or cell viability (21). Thus, the diverse roles of mitochondrial dynamics in tumorigenesis are cell-type specific and may be dictated by various physiological or pathological conditions (28).

Mitochondria are characterized not only as cellular powerhouses but also as signaling organelles. Generally, the outer mitochondrial membrane (OMM) is regarded as a platform where cell signaling pathways converge, while the inner mitochondrial membrane (IMM) is more responsible for mitochondrial respiration (19). Thus, we speculated that Drp1 regulated OMM fission might affect some cellular signaling pathways. Upregulation of total cellular STAT3, mitochondrial pSTAT3^S727^, and intranuclear pSTAT3^Y705^ was detected in GH3 cells when mitochondrial fission was inhibited by the Drp1 inhibitor Mdivi-1. Similar overexpression of the three proteins was observed in IGHPAs samples. Activation of STAT3 signaling is well recognized in several cancers and promotes tumor cell proliferation, survival, invasion and immunosuppression (41, 42). Transcription of MMP2/9 is directly regulated by STAT3 in several cancers, especially the phosphorylation of STAT3 at Tyr705 (27, 43, 44). In our study, we found that inhibition of STAT3 and pSTAT3^Y705^ significantly downregulated the expression of MMP2/9 and attenuated Mdivi-1-induced invasion of GH3 cell lines, PAPCs and MMQ cell lines, providing a probable mechanism by which STAT3 mediates mitochondrial hypofission induced invasion of IGHPAs. In addition to the canonical nuclear gene transcription regulation of STAT3, the role of noncanonical mitochondrial STAT3 signaling has been confirmed in tumorigenesis (42, 45). We found that inhibition of STAT3, but not pSTAT3^Y705^, significantly decreased the mitochondrial hypofission induced proliferation of GH3 cell lines, PAPCs and MMQ cell lines. Moreover, inhibition of STAT3, but not pSTAT3^Y705^, enhanced the mitochondria mediated apoptosis of GH3 cell lines. These data suggest a pro-proliferative role of mitochondrial pSTAT3^S727^ in GHPAs *via* anti-apoptosis. Mitochondrial pSTAT3^S727^ has been confirmed to regulate the activity of the electron transport chain, transcription of mtDNA and the mitochondrial permeability transition pore in several cell types, which could influence cellular proliferation by altering the production of ATP, ROS and mitochondrial transcripts (42, 45, 46). Ezzat et al. confirmed that fibroblast growth factor receptor 4 facilitates pituitary GH cell tumorigenesis *via* activation of mitochondrial pSTAT3^S727^, and further experiments concluded that mitochondrial pSTAT3^S727^ was a therapeutic target of pasireotide, which was approved for treating patients with GHPA (47, 48). Conclusively, mitochondrial hypofission in GHPA might enhance the invasion and proliferation of tumor cells *via* activation of intranuclear pSTAT3^Y705^ and mitochondrial pSTAT3^S727^, respectively.

In summary, this study revealed that mitochondrial hypofission caused by downregulation of Drp1 was responsible for the invasiveness and high proliferation of GHPAs. The underlying mechanisms might include activation of STAT3, which is especially dependent upon phosphorylation at S727 and Y705 in mitochondria and nuclei, respectively. Conclusively, our findings provide a new perspective on how mitochondria regulate the development of IGHPAs. However, whether the homozygous or heterozygous gene type is responsible for this alteration of Drp1, and the factors that trigger abnormal mitochondrial dynamics and consecutive dysregulation of mitochondrial metabolism in tumorigenesis requires further investigation.

## Data Availability Statement

The original contributions presented in the study are included in the article/**Supplementary Material**. Further inquiries can be directed to the corresponding authors.

## Ethics Statement

The collection procedure of patient tissue samples in this study was approved by laboratory animal welfare and ethics committee of Xinqiao Hospital (the ethical review number: 2018-049-012). The patients/participants provided their written informed consent to participate in this study.

## Author Contributions

YZ, HY, and SL designed the experiments. YZ and LZ performed the stereological experiments. YZ, KF, and ZZ carried out the cell experiment *in vitro*. YZ and YJG performed the *in vivo* experiments. YZ and XD carried out the immunohistochemical staining and analyzed the data. YZ, LZ, and SL wrote the manuscript. All authors read and approved the final manuscript.

## Funding

This work was supported by the Nursery Project of Army Medical University (No.2019R054),Natural Science Foundation of Chongqing (No.cstc2019jcyj-msxmX0475) and Basic Research Project of Army Military Medical University (2019XQN12).

## Conflict of Interest

The authors declare that the research was conducted in the absence of any commercial or financial relationships that could be construed as a potential conflict of interest.

The reviewer ZH declared a shared affiliation with one of the authors LZ, to the handling editor at the time of review.

## Publisher’s Note

All claims expressed in this article are solely those of the authors and do not necessarily represent those of their affiliated organizations, or those of the publisher, the editors and the reviewers. Any product that may be evaluated in this article, or claim that may be made by its manufacturer, is not guaranteed or endorsed by the publisher.

## Supplementary Material

Supplementary Figure 1
**(A–C)** Expression of Mfn1, Mfn2 and OPA1 mRNA levels were assessed by RT-qPCR in NIGHPAs (n = 12) and IGHPAs (n = 13) samples. **(D)** Representative electron microscopic images of mitochondria in four GH3 cell groups (VE, VE+Mdivi-1, Drp1^++/++^, Drp1^++/++^+Mdivi-1). ^#^P > 0.05. Scale bar = 0.5 um.

Supplementary Figure 2
**(A)** Cell cycle of GH3 cells in four groups (VE, VE+Mdivi-1, Drp1^++/++^, Drp1^++/++^+Mdivi-1) at 48 h were analyzed by flow cytometry (n = 3). **(B)**. Cell apoptosis at 48 h were analyzed by flow cytometry (n = 3). **(C)** Mitochondrial membrane potential of four GH3 cell groups were detected by flow cytometry using Rhodamine123 at 48 h (n = 3). **(D)** Reactive oxygen species (ROS) were detected by flow cytometry using DCF-DA fluorescence at 48 h (n = 3).

Supplementary Figure 3
**(A)** Cell cycle of GH3 cells in three groups (VE+Mdivi-1, VE+Mdivi-1+HO-3867, VE+Mdivi-1+Cry) at 48 h were analyzed by flow cytometry (n = 3). **(B)** Cell apoptosis at 48 h were analyzed by flow cytometry (n = 3). **(C)** Mitochondrial membrane potential of three GH3 cell groups were detected by flow cytometry using Rhodamine123 at 48 h (n = 3). **(D)** Reactive oxygen species (ROS) were detected by flow cytometry using DCF-DA fluorescence at 48 h (n = 3).

Supplementary Figure 4 The invasion and proliferation of MMQ and PAPCs were affected by Drp1 *via* STAT3. **(A)** MMQ cell invasion were evaluated by transwell assay when treated with Mdivi-1, Mdivi-1+HO-3867, Mdivi-1+Cry (n = 3). **(B)** Statistical analysis of the invasive MMQ cell number in the four groups. **(C)** The number of MMQ cells in four groups were assessed by CCK-8 assay (n = 3). **(D)** PAPCs cell invasion were evaluated by transwell assay when treated with Mdivi-1, Mdivi-1+HO-3867, Mdivi-1+Cry (n = 5). **(B)** Statistical analysis of the invasive PAPCs cell number in the four groups. **(C)** The number of PAPCs cells in four groups were assessed by CCK-8 assay (n = 5). *P < 0.05, ***P < 0.001. Data were expressed as mean ± SEM.
