# Supplementary figures and images for: Drp1 Regulated Mitochondrial Hypofission Promotes the Invasion and Proliferation of Growth Hormone-Secreting Pituitary Adenomas via Activating STAT3
Source: Front Oncol. 2022 Apr 7;12:739631. doi: 10.3389/fonc.2022.739631 (PMC9021862; doi:10.3389/fonc.2022.739631)

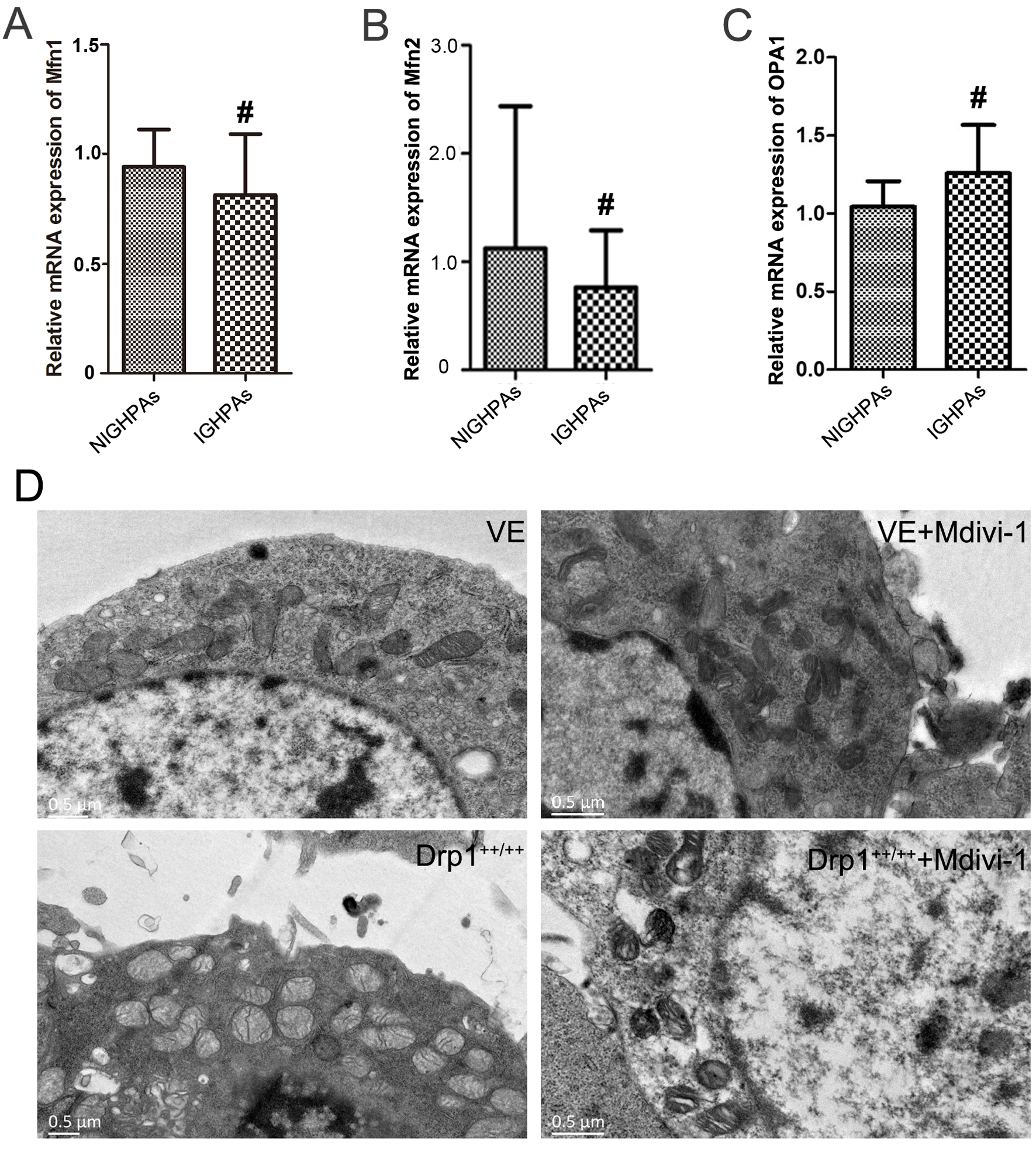

Supplement: Supplementary Figure 1 — (A–C) Expression of Mfn1, Mfn2 and OPA1 mRNA levels were assessed by RT-qPCR in NIGHPAs (n = 12) and IGHPAs (n = 13) samples. (D) Representative electron microscopic images of mitochondria in four GH3 cell groups (VE, VE+Mdivi-1, Drp1++/++, Drp1++/+++Mdivi-1). #P > 0.05. Scale bar = 0.5 um. [file Image_1.tif]

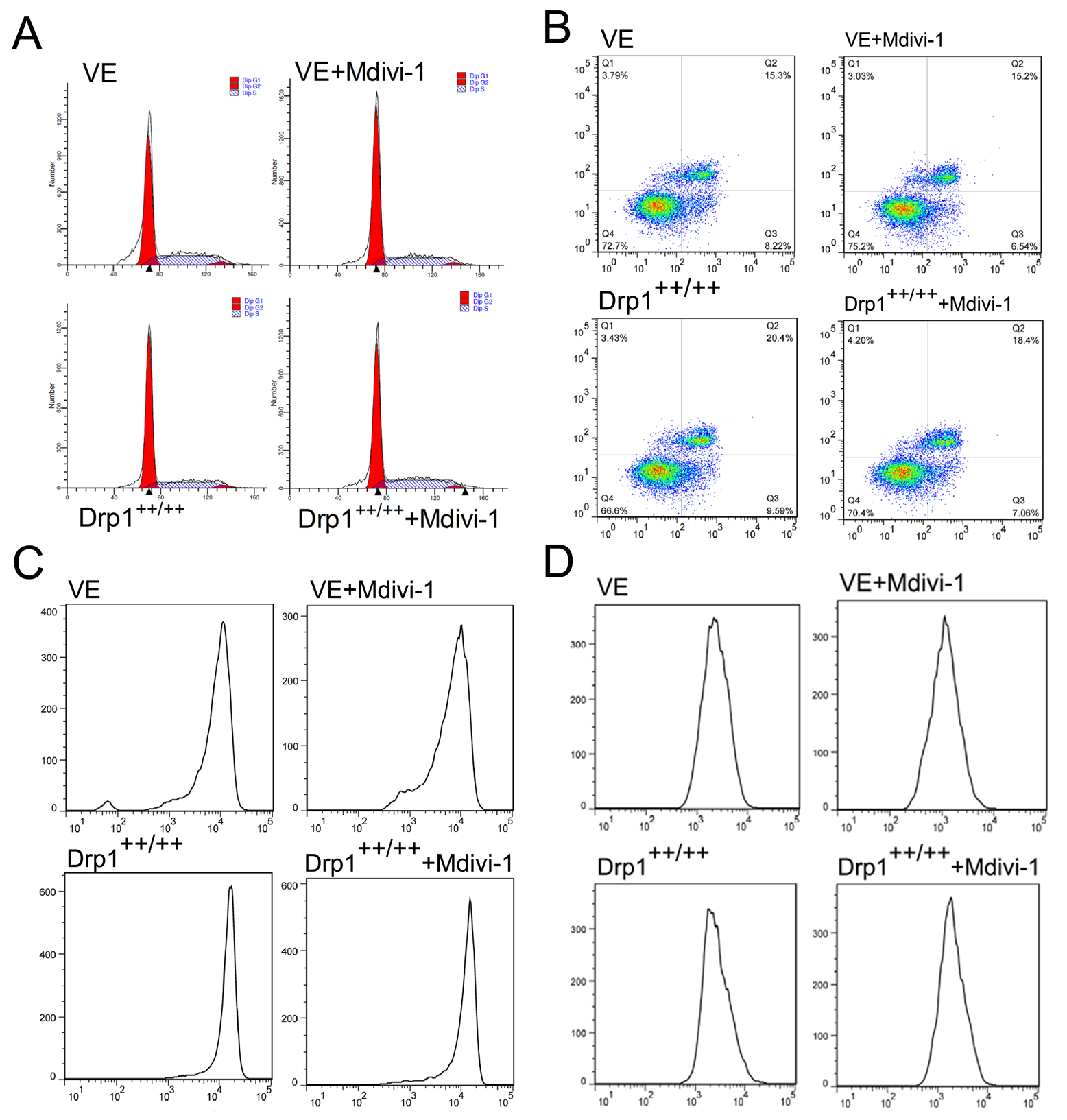

Supplement: Supplementary Figure 2 — (A) Cell cycle of GH3 cells in four groups (VE, VE+Mdivi-1, Drp1++/++, Drp1++/+++Mdivi-1) at 48 h were analyzed by flow cytometry (n = 3). (B). Cell apoptosis at 48 h were analyzed by flow cytometry (n = 3). (C) Mitochondrial membrane potential of four GH3 cell groups were detected by flow cytometry using Rhodamine123 at 48 h (n = 3). (D) Reactive oxygen species (ROS) were detected by flow cytometry using DCF-DA fluorescence at 48 h (n = 3). [file Image_2.tif]

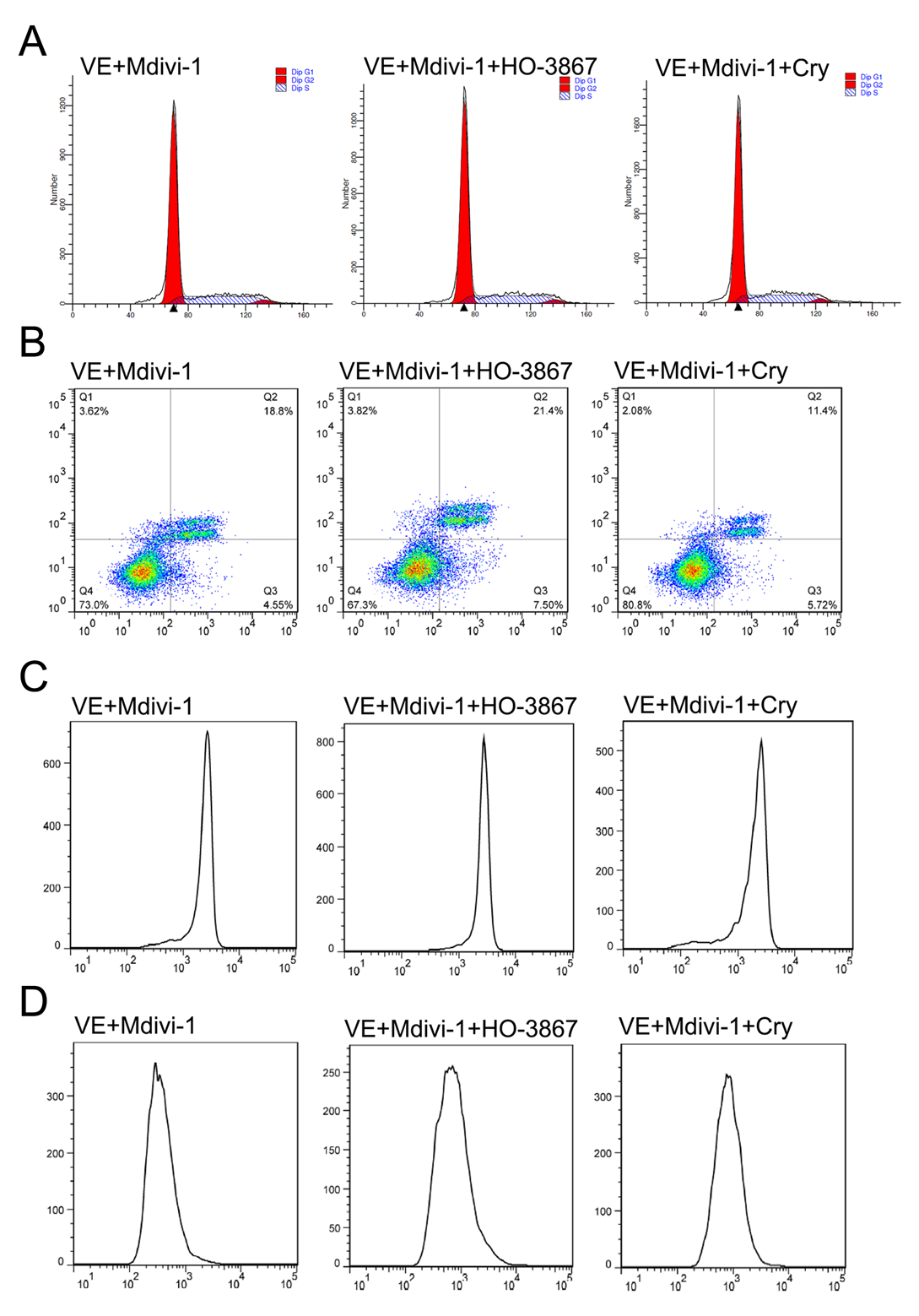

Supplement: Supplementary Figure 3 — (A) Cell cycle of GH3 cells in three groups (VE+Mdivi-1, VE+Mdivi-1+HO-3867, VE+Mdivi-1+Cry) at 48 h were analyzed by flow cytometry (n = 3). (B) Cell apoptosis at 48 h were analyzed by flow cytometry (n = 3). (C) Mitochondrial membrane potential of three GH3 cell groups were detected by flow cytometry using Rhodamine123 at 48 h (n = 3). (D) Reactive oxygen species (ROS) were detected by flow cytometry using DCF-DA fluorescence at 48 h (n = 3). [file Image_3.tif]

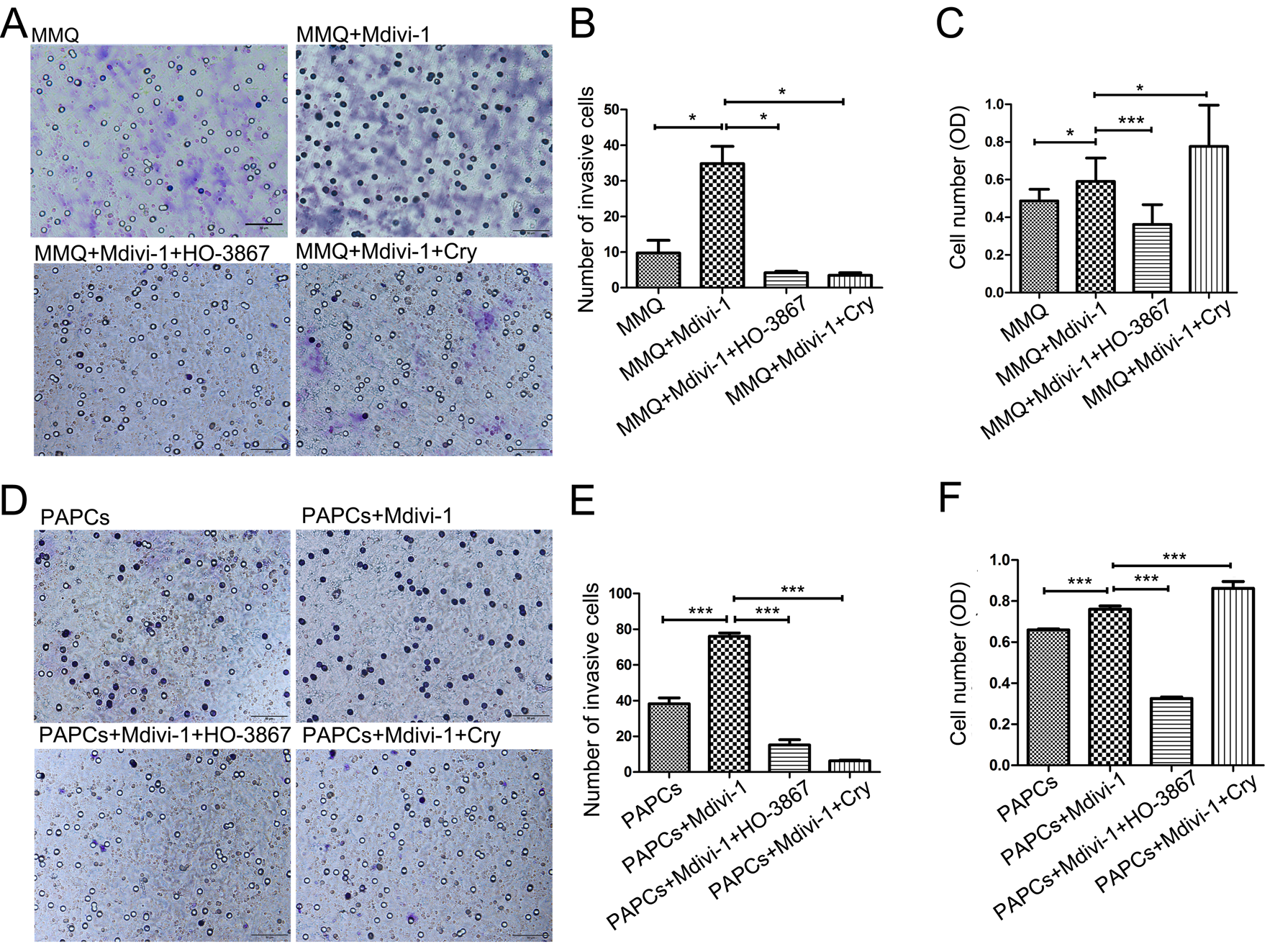

Supplement: Supplementary Figure 4 — The invasion and proliferation of MMQ and PAPCs were affected by Drp1 via STAT3. (A) MMQ cell invasion were evaluated by transwell assay when treated with Mdivi-1, Mdivi-1+HO-3867, Mdivi-1+Cry (n = 3). (B) Statistical analysis of the invasive MMQ cell number in the four groups. (C) The number of MMQ cells in four groups were assessed by CCK-8 assay (n = 3). (D) PAPCs cell invasion were evaluated by transwell assay when treated with Mdivi-1, Mdivi-1+HO-3867, Mdivi-1+Cry (n = 5). (B) Statistical analysis of the invasive PAPCs cell number in the four groups. (C) The number of PAPCs cells in four groups were assessed by CCK-8 assay (n = 5). *P < 0.05, ***P < 0.001. Data were expressed as mean ± SEM. [file Image_4.tif]
